# Heterotrophy and symbiosis affect energy reserves for pedal lacerates in the sea anemone *Exaiptasia diaphana*

**DOI:** 10.7717/peerj.20851

**Published:** 2026-02-25

**Authors:** Erick White, Maria Ruggeri, Virginia M. Weis

**Affiliations:** Integrative Biology, Oregon State University, Corvallis, OR, United States of America

**Keywords:** Symbiosis, Aiptasia, Laceration, Energetics, Asexual reproduction, Cnidaria, Symbiodiniaceae

## Abstract

Nutrient exchange between corals and their dinoflagellate symbionts is the foundation of the stable symbiosis that underpins coral reef ecosystem success. The cnidarian-dinoflagellate holobiont engages in both autotrophy (photosynthates supplied by the symbiont) and heterotrophy (feeding by the host on microscopic organisms and particulate matter) to meet their nutritional demands. While considerable research has been devoted to understanding nutrient dynamics in adult corals and other symbiotic cnidarians, less is known about how the combination of heterotrophy and autotrophy influences nutrition within and across generations. We investigated the role of symbiosis and heterotrophy in the sea anemone *Exaiptasia diaphana* (commonly called Aiptasia), a model system for the study of coral symbiosis. We examined how different feeding regimens affected parental growth and how nutritional status of the adult influenced nutrition of asexual offspring (pedal lacerates). After one-month, heterotrophic feeding resulted in larger pedal disk sizes in aposymbiotic adults regardless of lighting. However, in symbiotic groups, a lack of heterotrophy and/or autotrophy resulted in almost no growth or a decrease in body size. This suggests that symbiosis incurs a cost on the host when it is deprived of multiple food sources, and that autotrophy needs to be paired with heterotrophy for significant growth to occur in symbiotic adults. In pedal lacerates, we found that heterotrophic feeding and symbiotic state have an interactive effect on metabolite abundance. Symbiotic lacerates with access to food and light had significantly greater carbohydrates compared to all other groups, suggesting that the symbionts require both to produce carbohydrates in high quantities. Lipid content varied by symbiotic state, with aposymbiotic lacerates having more total lipids, while symbiotic lacerates had more nutrient-rich neutral lipids, indicating that symbiosis alters the production and abundance of different lipid classes. Symbiosis and heterotrophy significantly increased total protein in lacerates. Our results show that the combination of heterotrophy and autotrophy greatly increases growth rate and the abundance of carbohydrates and protein in symbiotic cnidarians, but nutritional lipids only differ based on symbiotic state, which suggests that the symbionts greatly increase the overall metabolic pool of the holobiont. In addition, our results show that there is a cost to hosting symbionts when autotrophy and/or heterotrophy are removed. This highlights the importance of heterotrophy in the success of symbiotic cnidarians within and across generations.

## Introduction

Scleractinian corals represent one of the most successful symbioses as evidenced by their ability to create large coral reef ecosystems that are home to a quarter of the planet’s marine biodiversity (Souter et al., 2020). The long-standing success of corals is in large part a result of the symbiosis formed between the cnidarian host and the endosymbiotic dinoflagellate algae from the family Symbiodiniaceae and the exchange of nutrients that occurs between the two (Pochon et al., 2006; LaJeunesse et al., 2018). While corals can meet almost 100% of their metabolic needs through the carbon acquired from symbiont-derived photosynthates (Muscatine et al., 1984), healthy corals also use heterotrophy to meet 15–35% of their daily metabolic demand (Grottoli, Rodrigues & Palardy, 2006). Heterotrophy has been shown to have positive effects on overall health and growth in both adult (Houlbrèque & Ferrier-Pagès, 2009; Rädecker & Meibom, 2023) and juvenile (Peng et al., 2020) symbiotic cnidarians. Having multiple sources of nutrient acquisition has allowed corals to succeed in oligotrophic, tropical environments and to build massive reefs systems by maintaining an efficient exchange of nutrients between partners and a regulation of symbiont density within the host.

Symbiont density within host gastrodermal cells is regulated to ensure that symbionts do not overpopulate the host and, likewise, that host growth does not outpace symbiont population growth (Muscatine et al., 1998). The cnidarian host possesses mechanisms to control the supply of nitrogen available to the symbiont, *via* reassimilation of host nitrogen and/or decreasing expression of nitrogen transporters between the host and symbiont, thereby regulating symbiont proliferation (Cui et al., 2019; Krueger et al., 2020; Xiang et al., 2020; Rädecker et al., 2023). Heterotrophy aids in this nitrogen-limiting mechanism by providing the host with another pool of nutrients to use, leading to a decrease in the expression of some nitrogen transporters between the host and symbiont (Xiang et al., 2020). This deficit in nitrogen triggers an increase in photosynthate transfer from the symbiont to the host, establishing a reciprocal nutrient exchange between partners that maintains the symbiotic relationship. Variations in environmental conditions can trigger an impairment in the nutrient exchange between symbiotic partners and result in varying levels of autotrophy and heterotrophy to survive and maintain the symbiotic relationship (Conti-Jerpe et al., 2020; Martinez, Grover and Ferrier-Pagès, 2024; Chei et al., 2025).

When corals experience a decrease in light levels, there is a shift from autotrophy to heterotrophy to compensate for the lack of photosynthesis, in addition to a decrease in symbiont density without total bleaching (loss of most or all symbionts brought on by stress) (Travaglione et al., 2023; Backstrom et al., 2024; Wang et al., 2024). Even when kept in the dark for months, studies have shown that symbionts can be maintained for long periods of time within host cells if the host is fed, and they can reproliferate when re-exposed to light (Jinkerson et al., 2022; Tran et al., 2024). Heterotrophy has also been shown to greatly enhance resilience to heat stress and aid in the recovery of corals after bleaching events (Grottoli, Rodrigues & Juarez, 2004; Grottoli, Rodrigues & Palardy, 2006; Rodrigues & Grottoli, 2007; Conti-Jerpe et al., 2020). However, when heterotrophy is absent from the system, symbiosis is more likely to break down and the host becomes more susceptible to damage. For example, a study in *Orbicella faveolata* showed that when exposed to heat stress without heterotrophy, symbionts increased their nutrient uptake and photosynthate production without concomitant transfer of photosynthate to the host. Meanwhile, the host experienced increased respiration rates and needed to use its own energy reserves without energy coming in from the symbiont (Baker et al., 2018). In *Exaiptasia diaphana*, when the juvenile hosts were starved over long periods of time, biomass dramatically decreased even to the point of death leaving apparently healthy symbionts behind (Peng et al., 2020). This indicates that symbiosis incurs a cost to the host during stress and highlights how the cnidarian-dinoflagellate symbiosis relies on both autotrophy and heterotrophy to survive changing environmental conditions. However, while there have been several studies that have examined nutrient dynamics in coral adults and juveniles separately, it is not yet clear how the nutrient dynamics experienced by adults influence the metabolic pool available in asexual offspring.

Previous studies that examined the effects of symbiosis and heterotrophy on energy reserves during sexual reproduction in the corals *Montipora capitata* and *Porites compressa* larvae have found that there are physiological tradeoffs during stressful environmental conditions. In these studies, carbon and nitrogen content in gametes was primarily derived from autotrophy, but nitrogen content of endosymbionts in adults was acquired heterotrophically when corals were bleached (Rodrigues & Padilla-Gamiño, 2022; Jaffe et al., 2023). In most species of corals, algae are not vertically transmitted in larvae, possibly because they present a physiological burden during early development (Baird et al., 2021), so instead they are supplied with different lipid compounds to fuel development (Buttari et al., 2024) until symbionts are horizontally acquired from the water column later in development. Asexual offspring, however, have vertically-transmitted symbionts within their tissue during formation, leading to a possible energy storage contribution from symbionts during asexual reproduction and development that has yet to be explored.

To investigate how the environmental conditions of the parent influence the metabolite pool of asexual offspring, we used the sea anemone *Exaiptasia diaphana* (commonly called Aiptasia) as a model system for the study of coral symbiosis (Rädecker et al., 2018; Jacobovitz, Hambleton & Guse, 2023). Aiptasia offers many advantages as a model for studying symbiosis such as its ability to be cultured with or without symbionts (aposymbiotic), the ease of propagating clonal lines naturally and artificially (Presnell, Wirsching & Weis, 2022), and the many molecular and omics-related resources available through the community (Baumgarten et al., 2015; Rosset et al., 2021; Mashini et al., 2024). Aiptasia can also rapidly reproduce asexually *via* pedal laceration whereby the adult anemone, referred to herein as a G0 parent, moves across a substrate and pinches off a small piece of the pedal disk (Hunter, 1984; Clayton, 1985; Clayton & Lasker, 1985). The resulting pedal lacerate, referred to herein as G1 offspring, then develops into a new juvenile polyp within a few days post-laceration (Schlesinger et al., 2010). While there have been studies that have examined how feeding, lighting, symbiotic state, and temperature influence G1 formation and development in Aiptasia (Hunter, 1984; Clayton, 1985; Schlesinger et al., 2010; Bedgood et al., 2020; Presnell, Wirsching & Weis, 2022), none have examined the nutrient dynamics that occur within and across generations of Aiptasia.

In this study, we used Aiptasia to investigate how symbiosis and different nutrient regimes experienced by G0s influence the composition of metabolites available to G1s from their parent. We hypothesized that (1) symbiotic G1s have a more abundant energy reserve compared to aposymbiotic G1s and (2) nutrient regimes experienced by G0s would impact the abundance of metabolites present in G1s. To date, our study is the first to examine the factors that influence energy reserves across generations in Aiptasia.

### Methods

### Animal maintenance

For all experiments, G0s from the H2 clonal line were used (Xiang et al., 2013). All animals were kept at 25 °C in a Percival incubator on a 12-hour/12-hour light/dark cycle, with a full spectrum irradiance of 10–20 µmol quanta m^−2^ s^−1^ and fed with S.presso (INVE Aquaculture, Belgium) gut-infused brine shrimp (*Artemia nauplii*). Symbiotic G0s were maintained with their native symbiont, *Breviolum minutum* (Xiang et al., 2013; LaJeunesse et al., 2018), inside clear polycarbonate tubs (Cambro) in 300 mL of 0.45 µm filtered artificial seawater (FASW; Instant Ocean). We generated aposymbiotic anemones *via* menthol bleaching, as described in Matthews et al. (2016). Animals were incubated in 300 mL of menthol-infused FASW (1.35 mL of 20 g/L menthol in 300 mL FASW) in black polycarbonate tubs with an air stone that ran for four hours a day for four consecutive days to mix and diffuse the menthol throughout the entire tub and ensure all anemones present were bleached of their symbionts. Confirmation of aposymbiotic status before the start of the experiment was determined by checking for presence of algae *via* chlorophyll autofluorescence under a Zeiss Axio Observer. A1 inverted microscope. Both symbiotic and aposymbiotic animals were maintained for at least three months prior to experiments.

### Generation, evaluation, and normalization of surgically-produced pedal lacerates

Adult symbiotic (*n* = 48) and aposymbiotic (*n* = 48) H2 G0s were transferred to polystyrene 6-well plates and starved for three days prior to generating G1s. G0s were then anesthetized in 7% MgCl_2_ for 15 min to make it easier to cut G1s from the adults. A #15 sterile scalpel was used to remove four to eight pieces of similarly sized pedal disk tissue per polyp (200–400 µm in diameter) until at least 150 were made (Presnell, Wirsching & Weis, 2022). G1s were placed individually into wells of a 48-well plate in fresh FASW for one to two days to allow the wound site to heal under the same lighting conditions described previously for symbiotic and aposymbiotic animals. After healing was complete, G1s were pooled into replicates of 20–30 each.

To evaluate protein as an independent metabolite for total energy reserves, total host lipids, carbohydrates, and protein values were normalized to the number of G1s pooled per replicate. Further analysis revealed that protein levels were influenced significantly by our treatment variables, potentially making them an unsuitable index for our results. Principal component analysis revealed that host protein concentrations and number of G1s per replicate were directionally related, indicating that number of G1s per replicate might be a suitable normalization alternative (Fig. S1). After performing linear regression model analysis comparing number of G1s per replicate to protein concentrations, we found that protein levels and lacerate number were significantly correlated to each other (Fig. S2; Linear model, *R*^2^ = 0.4139, *p* = 0.0001). When energy reserves were normalized to number of G1s per replicate, the major trends were not affected. Therefore, we determined that lacerate number made a suitable and effective index in lieu of protein for further analyses.

Naturally-produced G1s were also generated one to three days post-laceration (Supplemental Information 1) to compare the total energy reserves and algal density between naturally- and surgically-produced G1s to determine if surgically-produced G1s are a suitable substitute (Supplemental Information 1). Levels of total host carbohydrates (two-way analysis of variance (ANOVA), *p* = 0.3011), proteins (two-way ANOVA, *p* = 0.5614), and algal density (Student’s *t*-test, *p* = 0.8247) did not significantly differ between naturally- and surgically-produced G1s (Fig. S3–S5). We believe that there was no difference in the effect of size of G1s between natural and artificial G1s, because there was no significant difference in host protein concentration (Fig. S4). We did detect more total lipids in surgically-produced G1s compared to naturally-produced G1s (two-way ANOVA, *p* = 0.0001) in both symbiotic and aposymbiotic animals, although the patterning of symbiotic *vs* aposymbiotic animals between the two methods of lacerate generation were the same (aposymbiotic values were higher than symbiotic ones in both treatments) (Fig. S6 and Tables S1–2, Tukey HSD). Because of the similar patterning in lipid values and the absence of other differences in energy reserves or algal density between modes of G1 generation, we concluded that G0 adults do not provision lacerates differently than material that is surgically produced. Therefore, to generate large sample sizes and multiple replicates for experiments, we proceeded to use surgically-produced G1s for all subsequent experiments.

### Energy acquisition experiment

**Figure 1:**
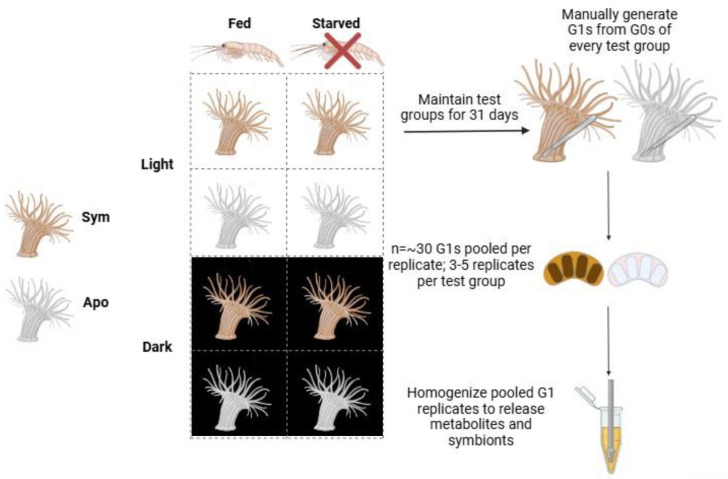
Experimental design of the energy acquisition experiment. Symbiotic (Sym) and aposymbiotic (Apo) G0 adults (*n* = 12 each) were randomly assigned to a treatment. For 1 month, anemones were subjected either to a 12:12 light:dark cycle and fed brine shrimp, a 12:12 light:dark cycle and starved, kept in 24 h of darkness and fed brine shrimp, or starved and kept in 24 h of darkness. At the end of the treatment, G1s were generated from G0s in each condition by cutting similar-sized pieces of tissue from the pedal disk (*n* = 150 total; *n* = 30 per replicate; five replicates). Figure made in BioRender.

Nutrient composition in G1s was quantified as a function of light, feeding regimen and symbiotic state of G0 parents to test the effects of parental nutrition and symbiosis on G1 energy reserves. We examined the interactions of symbiotic state (symbiotic *vs* aposymbiotic), feeding status (fed *vs* starved) and light regime (light *vs* dark to control for algal productivity) on G1 nutritional status (Fig. 1). Symbiotic and aposymbiotic G0s (*n* = 12 each) were divided into four feeding regimens differing in access to light and brine shrimp: (1) 12 hr L:D cycle and fed, (2) fed and kept in 24 hr darkness, (3) 12 hr L:D cycle and starved, and (4) kept in 24 hr darkness and starved (Fig. 1). All G0s selected for the experiment had a pedal disk diameter of at least three millimeters to ensure that they were mature adults. G0s were maintained in the same Percival incubator under the same light and temperature conditions as described in “*Animal husbandry”* and fed S.presso gut-infused brine shrimp three times a week for one month. Plates were cleaned and water was replaced with fresh FASW after feeding. Relative measurements of the pedal disk areas of G0s from the same animal were imaged on a Zeiss Axio Observer.A1 inverted microscope before and after treatment as a proxy for relative growth rate because it is non-invasive, positively correlates with dry weight measurements, and because growth in Aiptasia is body-size dependent and must be corrected at the individual level (Leal et al., 2012; Bedgood et al., 2020; Garschall et al., 2024). A microscope slide with a 10-micrometer scale was imaged and used to normalize a pixel to micrometer ratio for accurate measurements of pedal disk area. Images of pedal disk area from each animal on Day 0 and 31 were measured in the imaging software FIJI to track the effects of feeding regimen on host growth. Total change in pedal disk areas (μm^2^) in individual G0s was calculated by subtracting the initial (Day 0) measurement from the final (Day 31) measurement. Immediately following the one-month acclimation period for G0s, G1s were surgically generated as described above over the next three days until three to five replicates of pooled G1s (20–30 each) per condition were collected. Lacerates collected over each day of collection were evenly distributed among each replicate to eliminate any effect of timing of collection on the results. All samples were subjected to the total energy reserves protocol described below.

### Quantification of carbohydrates, lipids, and protein to determine total energy reserves

To quantify total concentrations of host lipids, carbohydrates, and protein, a total energy reserve protocol was adapted from techniques outlined in Baumann et al. (2021) for all three metabolites described here. Pooled G1 replicates were placed into new 1.5 mL microfuge tubes of 200 µL chilled RIPA extraction buffer (100 mM 7.33 pH Tris, 10 mM 8.04 pH EDTA, 100 mM NaCl) and protease inhibitor (Roche 04693159001) and homogenized with a motorized pestle. Samples were loaded onto a Qiagen TissueLyser II with a three mm diameter metal bead added to each tube and samples were lysed for 1 min at 3,000 Hz/min to shear cells and break up any remaining algal clumps. The resulting suspension was transferred to a fresh tube; original tubes were rinsed with chilled extraction buffer and added to the fresh tube to a total volume of one mL. Samples were centrifuged at 5,000 rpm for 5 min at 4 °C to pellet algae and other cellular debris. Only the soluble fraction was used for further analysis because it contains non-membrane-bound, host metabolites involved in energetic pathways, which was the primary interest for this study. The aliquots of the resulting extract were used for assays of total host lipids, carbohydrates, and proteins as described below. Algal pellets were stored at 4 °C for further analysis.

Carbohydrate concentration was quantified using a modified phenol-sulfuric acid assay. A 100 µL aliquot of the original extract or standard (see below) was added into a 1.5 mL microfuge tube containing 2.5 μL of 5% phenol. Tubes were vortexed for three sec followed by the addition of 250 μL of 98% sulfuric acid. Seven glucose standards (Sigma Aldrich G6918) ranging in concentration from 0–1 mg/mL were made by mixing one mg/mL glucose with MilliQ water. Samples and standards were incubated for 30 min in a dry bead bath at room temperature (RT) to allow for color development. Samples and standards were analyzed by aliquoting 100 μL in triplicate into a 96-well plate and read at 485 nm on a SpectraMax M3 plate reader using SoftMax Pro version 6.2 software. Standards were used to generate a standard curve to calculate concentrations of samples in micrograms.

Total lipid concentration (across all classes of lipids) was quantified using a modified sulfo-phospho-vanillin assay. Lipids were extracted from 600 µL aliquots of extract by adding 400 µL of chloroform and 200 µL methanol to the extract and incubated on a shaker for 20 min. Subsequently, 160 μL of 1X phosphate-buffered saline (PBS) was added to each tube and inverted to induce layer separation and then centrifuged at 3,000 rpm for 5 min. The bottom organic layer containing dissolved lipids was decanted and aliquoted into three 100 μL replicates in a 96-well polypropylene plate. Cholesterol standards (Millipore Sigma PHR1533) were aliquoted in triplicate ranging in value from 0–100 µg. Fifty μL of methanol was added to each well with sample or standard and incubated under a fume hood at 80 °C for 20 min in a dry bead bath until the solvent evaporated. One hundred μL of 98% sulfuric acid was added to each well, incubated at 80 °C for 30 min and then cooled on ice for 2 min. Fifty μL of 17% vanillin in phosphoric acid was aliquoted into each well of a fresh 96-well polystyrene plate. After 2 min, 75 μL of each sample and standard were added to the wells of the new plate and incubated at RT for 10 min to allow for color development. The plate was read at 540 nm on a SpectraMax M3 plate reader using SoftMax Pro version 6.2 software. Standards were used to generate a standard curve to calculate concentrations of samples in micrograms.

Protein concentrations were quantified using the Bradford assay (Bradford, 1976). Bovine serum albumin (BSA) standards (BioRad 500-0206) from 0 to 2 mg/mL were created by mixing 2 mg/mL BSA in radioimmunoprecipitation assay (RIPA) buffer. Five μL of standards and samples were added into a new 96 well plate followed by 250 μL of Coomassie stain and read at 595 nm on a SpectraMax M3 plate reader using SoftMax Pro version 6.2 software. Standards were used to generate a standard curve to calculate concentrations of samples in micrograms.

### Quantification of neutral lipids using Oil Red O

Neutral lipids are hydrophobic molecules with multiple, long carbon chains that can be densely packed together, which make them commonly used for energy storage (Lehmann, 2018). Examples of these molecules include triglycerides, wax esters, and cholesteryl esters. To test for neutral lipid localization and abundance, we conducted histological staining for neutral fatty acids and lipids that are used for energy with the lipophilic dye Oil Red O (ORO, Sigma Aldrich 00625) adapted from the protocol found in Yoganantharjah et al. (2017). G0s were kept in similar feeding and lighting conditions (symbiotic fed and starved G0s at 12:12 light-dark cycle and aposymbiotic fed and starved G0s in total darkness) for one month and G1s were surgically generated as described above. Sample G1s were first pooled into four or five replicates per test condition (15–20 per replicate) and placed into 1.5 mL microfuge tubes. Samples were rinsed twice with FASW to remove any residual debris from the well and then incubated in 7% MgCl_2_for 15 min before being fixed in 4% paraformaldehyde (PFA, Electron Microscopy Sciences 15710) overnight at 4 °C. PFA was removed from the tubes and samples were rinsed in 1X PBS twice for 10 min at RT. Next, samples were incubated in 60% isopropanol (Mallinckrodt 3043) at RT to permeabilize membranes. Samples were incubated in isopropanol until all chlorophyll pigment from symbiotic samples was removed (approximately 60 min) to ensure there was no background chlorophyll contamination during spectrophotometric analysis. Then, isopropanol was removed from the tubes and 100 μL of 0.3 g/mL ORO, dissolved in isopropanol, was added to each tube and incubated for two hours at RT. Afterwards, ORO was removed from the tubes and the tubes were washed with 1X PBS to remove any residual ORO from the tubes. Stained G1s were then removed from the tubes and placed on image slides where representative images from each test condition were captured on a Leica M165 FC dissecting stereomicroscope. G1s from every test condition were then placed into new 1.5 mL microfuge tubes and 100 μL 4% Igepal (Sigma Aldrich I8896) (made in 100% isopropanol) was added as a detergent to release the ORO dye incorporated into G1 tissue into solution. Fluid from each sample was removed and added to a 96-well plate with ORO standards ranging from zero to 0.3 g/mL in nuclease-free water (Life Technologies AM9937). Sixty percent isopropanol was used as a negative control. Concentration of dye per sample was quantified at 495 nm on a SpectraMax M3 plate reader using SoftMax Pro version 6.2 software. Standards were used to generate a standard curve and to calculate concentrations of samples in micrograms. Separate fixed symbiotic samples underwent the 60% isopropanol and 4% Igepal incubation steps, and the resulting solution was measured on the plate reader to confirm that there was no artifact of background contamination from chlorophyll.

### Algal density in host tissues

To quantify symbiont density in host tissues, preserved algal pellets from homogenized samples were resuspended in one mL of FASW. Ten microliters of algal suspension were placed onto counting chamber slides (Countess Invitrogen) and counted on a Countess II automated cell counter (Life Technologies) in triplicate to acquire a mean algal count per sample replicate.

### Statistical analyses

All analyses were completed in RStudio version 4.0.2 (R Core Team 2023). Data were tested for normality and homoscedasticity *via* Q-Q and residual plots; any datasets that did not meet these assumptions were log-transformed *via* the natural log function (ln) and reported as such. We used the *car* package to perform two-way ANOVAs (Fox & Weisberg, 2019) to calculate significant effects of single treatment and interactive effects (feeding condition * light condition * symbiotic state) on our measured traits. The *stats* package was used for Tukey honestly significant difference (HSD) post-hoc analyses and Student’s *t*-test for pairwise comparisons between all treatment groups. Statistical significance was set at *p*-values <0.05 (R Core Team, 2023). All figures were produced using the *ggplot2* package (Wickham, 2016).

## Results

### Source of nutrition affects growth in G0s and energy reserves in G1s

**Figure 2:**
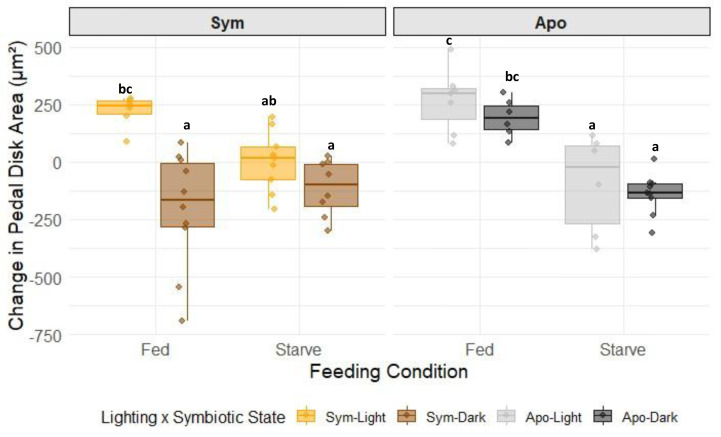
Overall change in relative pedal disk areas (µm^2^) of Aiptasia G0s (n = 8–12) subjected to differential feeding and light regimes between the beginning and end of treatment (31 days). Treatment groups are faceted out by symbiotic state, feeding condition, and lighting condition. Non-shared letters between treatment groups represent statistically significant differences in overall change in pedal disk size (Tukey HSD).

Feeding was the primary driver of changes to relative pedal disk size in G0s (Table S3, two-way ANOVA, *p* = 0.0001), with all fed groups, except for symbiotic G0s kept in dark, exhibiting an increase in relative pedal disk area between the start and end of the treatment (Fig. 2). Aposymbiotic G0 relative pedal disk size increased in those that were fed and decreased in those that were starved (Fig. 2 and Table S4, Tukey HSD). In symbiotic G0s, relative pedal disk size significantly increased in G0s that were fed and had access to light after 31 days. However, all other symbiotic groups either showed little growth or a decrease in relative pedal disk size after 31 days, but none of these three groups were significantly different from each other (Fig. 2 and Table S4, Tukey HSD). Because there was a significant difference in change in relative pedal disk size between symbiotic, fed G0s that either had access to light or were kept in the dark, there was an interactive effect of lighting with symbiotic state where having access to light had a greater effect on growth in symbiotic G0s compared to aposymbiotic G0s (Tables S3–S4; two-way ANOVA, *p* = 0.0156).

**Figure 3:**
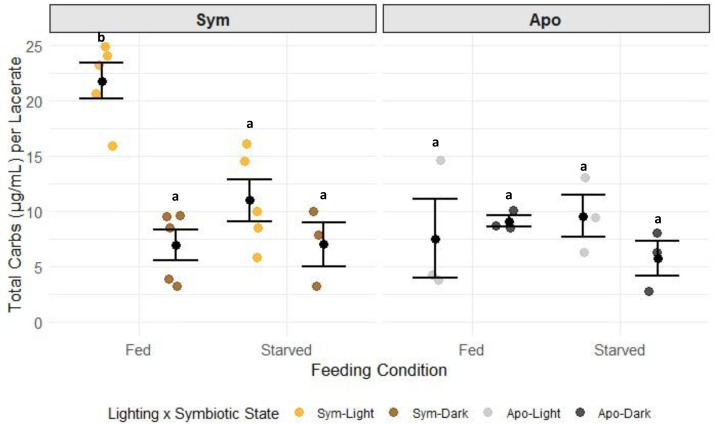
Total host carbohydrates per G1 by symbiotic state, lighting condition, and feeding condition. Black dots and error bars represent the mean concentration ± standard error of each group; colored, jittered dots represent individual replicate values within each group. Non-shared letters between treatment groups represent statistically significant differences in total carbohydrate concentration (Tukey HSD).

**Figure 4:**
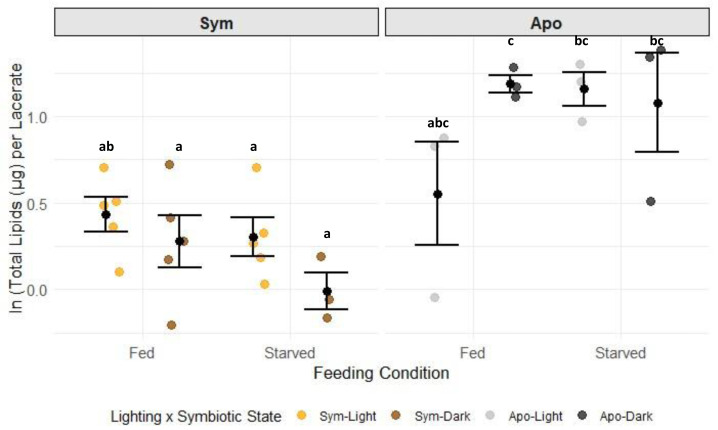
The natural log of total host lipids per G1 by symbiotic state, lighting condition, and feeding condition. Black dots and error bars represent the mean concentration ± standard error of each group; colored, jittered dots represent individual replicate values within each group. Non-shared letters between treatment groups represent statistically significant differences in total lipid concentration (Tukey HSD).

**Figure 5:**
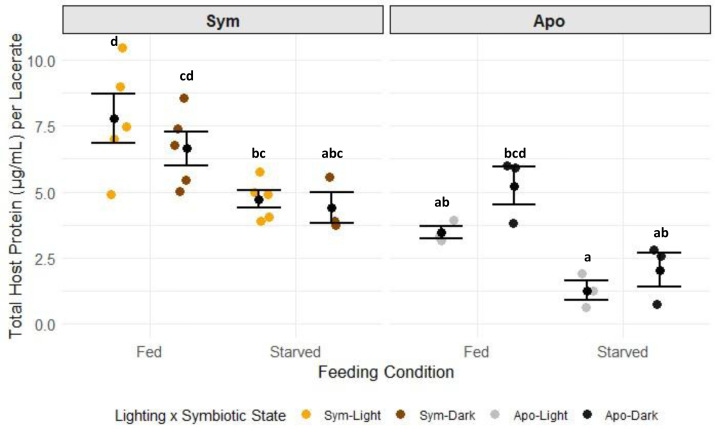
The concentration of total host protein per G1 by symbiotic state, lighting condition, and feeding condition. Black dots and error bars represent the mean concentration ± standard error of each group; colored, jittered dots represent individual replicate values within each group. Non-shared letters between treatment groups represent statistically significant differences in total host protein concentration (Tukey HSD).

Carbohydrate concentrations in G1s were only significantly different in the sym-fed-light test group (Fig. 3, Table S6, Tukey HSD), while all other test groups had similar levels of carbohydrates. The strong response in the sym-fed-light group resulted in a significant interactive effect of lighting condition, symbiotic state, and feeding condition on this dataset (*p* = 0.0081) (Fig. 3, Table S5, two-way ANOVA). Total lipids were primarily dependent on symbiotic state (Table S7; two-way ANOVA, *p* = 0.0001). Aposymbiotic G1s had higher concentrations of total lipids than their symbiotic counterparts (Table S8). There was also an interactive effect of lighting and symbiotic state (*p* = 0.0324) (Fig. 4, Table S7, two-way ANOVA), likely a result from the large, though not significant, difference in lipid concentration between apo-fed-light and apo-fed-dark G1s (Fig. 4, Table S8, Tukey HSD). There were significant effects of feeding condition (*p* = 0.0001) and symbiotic state (*p* = 0.0001) on the amount of protein detected in G1s (Table S9, two-way ANOVA). G1s that came from symbiotic-fed G0s had the highest amount of host protein, whereas aposymbiotic-starved had the least amount of protein (Fig. 5, Table S10). Total protein was similar between starved symbiotic groups and fed aposymbiotic groups (Table S10, Tukey HSD). While we did find a significant effect of lighting on symbiont density (two-way ANOVA *p* = 0.0146), as seen with G1s that came from G0s kept in the light generally having higher symbiont counts, none of the symbiont counts from any group were significantly different from each other (Fig. S7).

### Symbiosis and feeding leads to high neutral lipid storage

**Figure 6:**
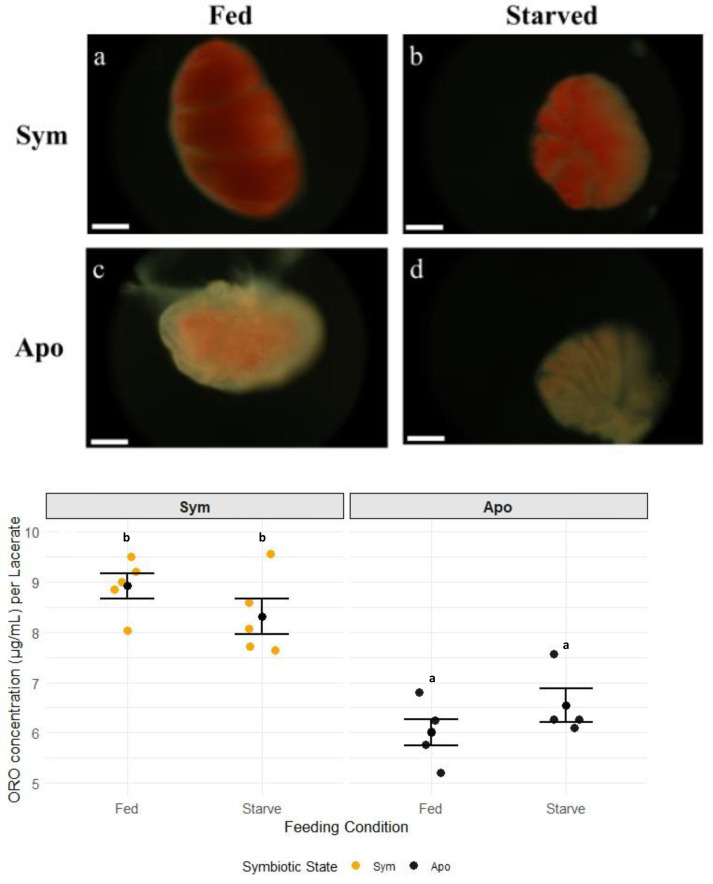
Oil Red O staining in Aiptasia G1s as a function of feeding and symbiotic state. (A–D) Representative images of Oil Red O (ORO) staining in G1s from each test condition one day post-laceration. Images show localization and intensity of staining in G1s from (A) sym-fed-light G0s, (B) sym-starved-light G0s, (C) apo-fed-dark G0s, and (D) apo-starved-dark G0s. The scale bar (bottom left corner) is 100 µm for all specimens. Bottom panel: concentration of neutral lipids stained by ORO per lacerate, based on symbiotic state and feeding. Black dots and error bars represent mean ± standard error for each group; colored dots represent individual replicate values (*n* = 4 or 5). Non-shared letters between treatment groups represent statistically significant differences (Tukey HSD).

Symbiotic state greatly influenced the concentrations of energy-rich lipid abundance in G1s (Fig. 5, Table S11; two-way ANOVA, *p* = 0.0001). Sym-fed-light G1s stained a dark red with ORO, indicative of a high neutral lipid concentration (Fig. 6A), and the stain was evenly spread throughout all tissue layers. Sym-starved-light G1s showed a lighter red coloration, but with a similar staining pattern (Fig. 6B). Apo-fed-dark G1s stained a light pink, primarily in the gastrodermal tissue (Fig. 6C), while G1s from apo-starved-dark G0s showed almost no stain in any tissue (Fig. 6D). Quantification of ORO showed significantly higher dye present in symbiotic compared to aposymbiotic animals (Fig. 6, Table S12, Tukey HSD). Based on our two methods of lipid quantification, it appears that the effect of symbiosis on lipids in G1s varies based on the class of lipid that is measured. Total lipids, measured using the sulfo-phospho-vanillin assay, were significantly higher in aposymbiotic than in symbiotic G1s with no effect from feeding or lighting detected. In contrast, when measuring neutral, nutrient-rich lipids through ORO staining, we found a significantly higher concentration of lipids in symbiotic than in aposymbiotic G1s.

## Discussion

### Heterotrophy and autotrophy are required for growth and metabolite storage in symbiotic G0s

Although heterotrophy alone led to growth in adults, as seen in the fed aposymbiotic G0s, symbiosis alone did not result in growth in adults. In symbiotic adults, only the combination of autotrophy and heterotrophy increased relative pedal disk size, suggesting that Aiptasia relies on a mixotrophic nutritional strategy (Fox et al., 2018; Radice et al., 2019). While photosynthates can supply the host with the majority of its energetic needs (Muscatine et al., 1984), feeding has proven to be especially important in fueling growth in symbiotic corals. Fed corals have been shown to have at least double the amount of organic matrix and calcification rates compared to starved animals, resulting in more overall growth (Ferrier-Pagè et al., 2003; Houlbrèque, Tambutté & Ferrier-Pagès, 2003; Houlbrèque et al., 2004a; Tremblay et al., 2016). For *Stylophora pistillata,* the addition of heterotrophic feeding greatly increased rates of gross photosynthesis, host respiration, and energy storage into host tissues (Tremblay et al., 2016). Heterotrophic feeding, therefore, could have enhanced autotrophy and subsequent photosynthate transfer in our symbiotic-fed animals. Another study on *Montipora capitata* and *Porites compressa* found that heterotrophically- and autotrophically-acquired energy were metabolized at different rates in symbionts and host tissue (Rodrigues & Padilla-Gamiño, 2022). It is possible that there are differences in metabolism between autotrophic and heterotrophic food sources in Aiptasia that require the combination of autotrophy and heterotrophy in symbiotic anemones to generate enough energy to store and use for growth. Future studies with isotopic labeling of carbon are needed to determine if heterotrophy is acting as an additive or synergistic force on the autotrophic production and transfer of photosynthates to the host. Our findings in Aiptasia highlight the importance of heterotrophic feeding by the host for the maintenance of host biomass and symbiosis under changing environmental conditions. However, it is important to note that the energy budgets between sea anemones such as Aiptasia and scleractinian corals likely differ due in part to the resources needed for the production and maintenance of the coral skeleton. Future studies could examine how much energy is acquired by autotrophy and heterotrophy individually and combined, and how that energy is allocated in anemones *vs* corals.

### The absence of heterotrophy and/or autotrophy results in a cost to maintaining symbiosis

In symbiotic anemones without access to light, we found that there was a significant reduction in host biomass compared to treatments that had access to both light and food (Fig. 2). The reduction in mean relative pedal disk size of sym-dark G0s after 31 days was similar to what was observed in apo-starved G0s, suggesting that there is a cost to symbiosis when autotrophy is inhibited that is not offset by heterotrophy. These findings align with a similar study of other symbiotic cnidarians that showed that when photosynthesis was inhibited, symbionts were able to be maintained in the host either at similar or lower levels than at the start of treatment (Jinkerson et al., 2022). It is therefore possible that the host used its own nutrient stores to maintain symbionts in the absence of reciprocal nutrient exchange from symbionts. In addition, Symbiodiniaceae can rely on heterotrophic food sources to survive (Steen, 1987; Jeong et al., 2012; Xiang et al., 2018), so they could have been parasitizing the host to maintain their own metabolic functions. However, our findings that symbiont density did not change significantly based on lighting or feeding (Fig. S7) corroborates those in Jinkerson et al. (2022) that the nutrition symbionts may have parasitized from the host was not enough to allow for proliferation of symbionts.

The observation that relative pedal disk size did not change in sym-starved G0s in the light over one month suggests that autotrophy alone is able to maintain body size but not growth in Aiptasia (Fig. 2). Similarly, a previous study in chronically starved adult Aiptasia showed that there was a continued transfer of photosynthates from symbiont to host, facilitated by a significant reduction in host biomass and algal density, that was sufficient to meet the basal metabolic demand of the host (Rädecker & Meibom, 2023). Our G0s may also have been acquiring nutrition to maintain body size by digesting some of their symbionts. Starved scleractinian corals have been shown to feed on symbionts to access nitrogen and phosphorous pools to fuel growth (Wiedenmann et al., 2023). It is possible that the lower, though not significant, algal density we observed in sym-starved-light G0s represents a similar consumption of algae (Fig. S7). Another explanation for the lower algae present and possible lower energy available is that the light levels in our incubator were kept at levels known to reduce algal density and gross photosynthesis in symbionts in Aiptasia, but encourage more symbiont nitrogen assimilation, which could lead to less transfer of photosynthates (Rädecker et al., 2023). However, further experiments would be needed to confirm that this phenomenon also occurs in G1 energy dynamics.

Similar studies in juvenile Aiptasia and pedal lacerates showed the opposite trend regarding symbiosis in the absence of heterotrophy. Symbiotic animals that were starved over long periods of time showed a decline in the health of the host that ultimately resulted in death, but with no reduction in algal population (Peng et al., 2020; Bedgood, Plichon & Weis, 2024). This suggests that heterotrophy takes on a larger role in maintaining symbiosis and host health depending on the life stage of the organism. While autotrophy alone may offset the energetic cost of symbiont maintenance and meet basal metabolic functions in adults, this study suggests that heterotrophy is required for survival and the success of the symbiosis in younger hosts. Sym-starved G1s kept in the light in our study had lower levels of carbohydrates, lipids, and protein compared to those using both autotrophy and heterotrophy (Figs. 3–6). It is possible that symbiosis is energetically costly to maintain in young hosts without heterotrophy, resulting in a lack of nutrients to support both the host and the symbiont population.

### Aiptasia do not provision asexual offspring for development

We found that only lipids were differentially abundant between natural and artificial G1s, with artificial G1s having a higher amount of total lipids than natural G1s. This suggests that nutrients are not provisioned for development in asexual offspring of Aiptasia, which contradicts previous research into nutrient provisioning of larval offspring for development. Coral and Aiptasia larvae are lecithotrophic, meaning that eggs are provisioned with maternally-derived carbon in the form of lipid droplets. After fertilization, planula larvae rely on these stores to fuel their metabolic needs through development until symbionts are acquired or heterotrophic food sources become available (Marlow & Martindale, 2007; Voss et al., 2023; Buttari et al., 2024). It is hypothesized that pedal lacerates rely on nutrients stored in the mesenteries of Aiptasia pedal disks from which they are formed until a food source becomes available (Presnell, Wirsching & Weis, 2022). Our results suggest that the energy reserves of G1s reflect the nutrients present in G0 pedal disks during laceration and not an intentional provisioning for asexual offspring.

We also observed that natural G1s have fewer total lipids than artificial G1s immediately post-laceration. This suggests that the process of naturally producing G1s depletes the reserve of total lipids in the offspring. The starlet sea anemone *Nematostella vectensis* has become an established model system for studying the mechanisms involved in development and regeneration. A study in *Nematostella* explored the molecular mechanisms underlying different developmental trajectories, namely embryogenesis, asexual reproduction by fission, and regeneration following injury. They found differences in expression of two of the seven investigated genes for developmental trajectory between transverse fission and regeneration (Burton & Finnerty, 2009). It is possible that the act of manually-generating G1s in Aiptasia reflects more of a regeneration-post-injury developmental trajectory and that it requires less energy to heal a wound site than to naturally produce G1s *via* pedal laceration. Future studies could repeat this experiment to examine the molecular pathways present in artificial and natural G1 production in Aiptasia.

### Metabolite pools are differentially affected by symbiosis, autotrophy, and heterotrophy

We found that feeding regimens experienced by G0s differentially affected metabolite abundance in G1s in the following ways: only sym-fed G1s in the light were able to retain significantly higher levels of carbohydrates, lipid abundance varied between symbiotic and aposymbiotic G1s based on the type of lipid measured, and protein content was higher in samples that were fed than those that were starved (Figs. 3–6). These results suggest that the presence or absence of symbiosis, autotrophy, and heterotrophy affects metabolism in unique ways.

When symbiotic G0s had access to both food and light and thus received nutrition *via* both heterotrophy and autotrophy, resulting G1s had a significantly greater reserve of carbohydrates than all other treatments (Fig. 3). This suggests that the combination of heterotrophy and autotrophy resulted in an excess of carbohydrates that were stored for later use, while in the other treatment groups, carbohydrates were metabolized. Symbionts are maintained in a nitrogen-limited state by the host not only as a means of controlling symbiont proliferation but also encouraging the transfer of photosynthates from the symbiont to the host (Muscatine & Porter, 1977; Falkowski et al., 1984; Cui et al., 2023; Rädecker et al., 2023). Because symbionts are not in direct contact with the surrounding seawater, access to nitrogen is dependent on what is available and accessible within the host. The host can passively modulate how much nitrogen is available through assimilating and recycling ammonium and producing it *via* its glutamate metabolism (Rädecker et al., 2021; Cui et al., 2022). Therefore, most of the nitrogen required for cellular processes and protein synthesis is acquired by heterotrophic feeding by the host, which meets much of the nitrogen requirement for the entire holobiont (Houlbrèque & Ferrier-Pagès, 2009). Also, the addition of pyruvate, an exogenous carbon source, was shown to enhance nitrogen assimilation in Aiptasia (Rädecker et al., 2023). Our results could be showing a similar effect that pairing heterotrophy with autotrophy results in higher carbohydrate reserves in G1s. However, future studies with isotopic labeling of carbon and nitrogen are needed to determine exactly how much photosynthate is transferred and incorporated between symbiotic partners and if nitrogen limitation is in fact present during G1 development. Heterotrophic feeding also had a greater influence on protein concentrations in the host as nitrogen is a key component of protein synthesis, evident with our fed animals having greater concentrations of protein than starved ones (Fig. 5). Rädecker & Meibom (2023) also showed that heterotrophic feeding influenced protein levels in Aiptasia, with starvation leading to severe drops in host protein levels. Studies on corals have also shown the positive effects of heterotrophy on sexual reproduction including gametogenesis (Gori et al., 2013; Jaffe et al., 2023) and larval survival (Viladrich et al., 2025). Our observations in asexual offspring align with these findings in adults and sexual offspring and show how heterotrophy can greatly influence metabolite pools.

Our results on lipid concentrations varied depending on the type of lipid we measured. Whereas our study found that aposymbiotic animals had higher concentrations of total lipids than symbiotic animals (Fig. 4), studies in *Montipora capitata* and *Porites compressa* found that bleaching caused a decrease in total lipids (Grottoli, Rodrigues & Palardy, 2006; Rodrigues & Grottoli, 2007). This difference could be due to both the stress related to bleaching and the facultative nature of the symbiotic state in Aiptasia compared to corals. In our study, the anemones had been bleached months prior to the beginning of the experiment and maintained as aposymbiotic animals. Therefore, the stress of bleaching was separated in time from the start of the experiment. Furthermore, the very fact that Aiptasia can be maintained in an aposymbiotic state indefinitely sets it apart from corals, which largely cannot survive long in a bleached state. In contrast to the total lipid assay, the ORO assay, which detects nutrient-rich neutral lipids, found that symbiosis had a significant and positive effect on neutral lipid concentrations (Fig. 6). These results align with studies that examined the lipidomic profile of the coral *Turbinaria reniformis* as a function of lighting and feeding. The authors of these studies found that there were significantly higher concentrations of storage lipids (wax esters, triglycerides) in symbiotic animals that were fed than those that were starved (Treignier et al., 2008; Tolosa et al., 2011). Our symbiotic animals had higher ORO concentrations than our aposymbiotic ones, and we did observe a trend, though not significant, that heterotrophic feeding increased ORO staining even more. Our results from the two different lipid assays suggest that the symbiotic state of Aiptasia has a significant effect on the production, consumption, and abundances of lipids that are present within the host. However, our ORO assay did not examine symbiotic animals kept in the dark and aposymbiotic animals kept in the light. Therefore, our conclusion that symbiotic state drives higher energy-rich lipid production could be a result of the lighting condition. It is possible that symbiotic animals kept in the dark would catabolize lipid stores to make up for the lack of photosynthate production. Also, aposymbiotic animals kept in the light might have a chance to reacquire symbionts or be influenced by microbial nutrient cycling affected by light. Future studies should repeat this experiment with a full factorial experimental design to determine if there is a combinatorial effect of symbiosis and lighting. In addition, a lipidomics study of symbiotic and aposymbiotic Aiptasia found that there were differences in concentrations of lipid classes based on the symbiotic state of the holobiont (Garrett et al., 2013). The authors suggested that the differences were not simply based on the combination of symbiont and anemone lipidomic profile, but rather that there were specific changes in the types of lipids produced depending on whether symbionts were present in hosts. Additional analyses such as LC/MS could distinguish between the lipid profiles of symbiotic and aposymbiotic G1s in comparison to adults and sexual offspring.

In summary, our findings suggest that Aiptasia only grows when the holobiont functions as a mixotroph, obtaining nutrition from both algal symbiont photosynthesis and heterotrophic feeding. However, when one of these food sources is absent from the symbiotic animal, it can have negative physiological consequences on the holobiont. Our data also suggest that metabolite pools are differentially affected by nutritional sources. Aiptasia, as a facultatively symbiotic organism, serves as an excellent model system to evaluate the independent effects of symbiosis, autotrophy, and heterotrophy on metabolite pools in asexual offspring. Future projects could expand upon this research by determining the identity of specific metabolites affected by autotrophy and heterotrophy, and how these nutrient stores affect and change during G1 development. While not examined in this study, there is the potential for the influence of host and symbiont microbiomes on energy reserves. Studies in Aiptasia have revealed that microbiomes differ based on symbiotic state and change the abundance of biogenic volatile organic compounds (BVOCs), which are indicators of metabolic processes in the host (Röthig et al., 2016; Costa et al., 2021; Wuerz et al., 2022; Wuerz et al., 2023). Microbial symbionts have also been shown to contribute to the transfer of lipids to the host in corals (Sikorskaya, Ermolenko & Efimova, 2022). Future studies could examine the contributions of microbial symbionts to energy reserves in Aiptasia under changing environmental conditions.

## Supplemental Information

10.7717/peerj.20851/supp-1 Supplemental Information 1 Supplemental Methods and Results

10.7717/peerj.20851/supp-2 Supplemental Information 2 PCA plot of the directionality and relationship of each metabolite variable and sample size (lacerates per replicate) in relation to test groups Abbreviations for test groups are as follows: AFD, aposymbiotic-fed-dark; AFL, aposymbiotic-fed-light; ASD, aposymbiotic-starved-dark; ASL, aposymbiotic-starved-light; SFD, symbiotic-fed-dark; SFL, symbiotic-fed-light; SSD, symbiotic-starved-dark; SSL, symbiotic-starved-light

10.7717/peerj.20851/supp-3 Supplemental Information 3 Linear model regression analysis of the correlation of sample size (lacerates) per replicate to host protein Y=26.5847 + 0.0206x, R^2^ = 0.4139, *p* = 0.0001. Abbreviations for test groups are as follows: AFD, aposymbiotic-fed-dark; AFL, aposymbiotic-fed-light; ASD, aposymbiotic-starved-dark; ASL, aposymbiotic-starved-light; SFD, symbiotic-fed-dark; SFL, symbiotic-fed-light; SSD, symbiotic-starved-dark; SSL, symbiotic-starved-light.

10.7717/peerj.20851/supp-4 Supplemental Information 4 Concentration of natural log transformed total host carbohydrates per lacerate in artificial and natural G1s based on symbiotic state Black dots and error bars represent group mean +/- standard error, while colored, jittered dots represent individual replicate (*n* = 30 G1s) optical density values optical run in triplicate. No significant differences were detected between comparisons (Tukey HSD).

10.7717/peerj.20851/supp-5 Supplemental Information 5 Concentration of total host protein per lacerate in artificial and natural G1s based on symbiotic state Black dots and error bars represent the mean concentration +/- standard error for each test group, while colored, jittered dots represent individual replicate (*n* = 30 G1s) optical density values optical run in triplicate. No significant differences were detected between comparisons (Tukey HSD).

10.7717/peerj.20851/supp-6 Supplemental Information 6 Algal density per lacerate in artificial and natural G1s Black dots and error bars represent the group mean +/- standard error in algal counts between five biological replicates of *n* = 30 G1s run in triplicate (shown in jittered, colored dots). No significant differences were detected between treatment groups (Student’s two-sample *t*-test).

10.7717/peerj.20851/supp-7 Supplemental Information 7 Concentration of total host lipids per lacerate in artificial and natural Aiptasia G1s as a function of symbiotic state Black dots and error bars represent group mean +/ standard error, while colored, jittered dots represent individual replicate (*n* = 30 G1s) optical density values optical run in triplicate. Different letters represent significant pairwise comparisons between test groups differences (Tukey HSD).

10.7717/peerj.20851/supp-8 Supplemental Information 8 Algal density per lacerate in symbiotic G1s that were either fed or starved and kept in the light or in the dark Black dots and error bars represent the mean concentration +/- standard error for each test group, while colored, jittered dots represent individual biological replicates (*n* = 30 G1s) of algal counts run in triplicate. No significant differences were detected between comparisons (Tukey HSD post-hoc analysis).

10.7717/peerj.20851/supp-9 Supplemental Information 9 Two-way ANOVA of the single and interactive effects of type of G1 (natural *vs.* artificial) and symbiotic status (sym *vs.* apo) on total host lipids Statistically significant values are bolded.

10.7717/peerj.20851/supp-10 Supplemental Information 10 Tukey HSD pairwise comparisons between treatment groups on the difference in total lipid concentrations based on type of G1 and symbiotic state Abbreviations: AA, artificial and aposymbiotic; AS, Artificial and symbiotic; NA, natural and aposymbiotic; NS, natural and symbiotic. Bolded values indicate significantly different p-values (*p* < 0.05).

10.7717/peerj.20851/supp-11 Supplemental Information 11 Two-way ANOVA of the single and interactive effects of feeding, lighting, and symbiotic state on the change in G0 pedal disk diameter Bolded values indicate statistical significance.

10.7717/peerj.20851/supp-12 Supplemental Information 12 Tukey HSD pairwise comparisons between treatment groups on the change in G0 pedal disk diameter after one month of exposure to different feeding regimens Bolded values indicate significantly different p-values. Abbreviations: AFD, apo-fed-dark; AFL, apo-fed-light; ASD, apo-starved-dark; ASL, apo-starved-light; SFD, sym-fed-dark; SFL, sym-fed-light; SSD, sym-starved-dark; SSL, sym-starved-light.

10.7717/peerj.20851/supp-13 Supplemental Information 13 Two-way ANOVA of the single and interactive effects of feeding, lighting, and symbiotic state on total host carbohydrates Bolded values indicate statistical significance.

10.7717/peerj.20851/supp-14 Supplemental Information 14 Tukey HSD pairwise comparisons between treatment groups on the mean of concentration of total host carbohydrates based on different feeding regimens Bolded values indicate significantly different p-values. Abbreviations: AFD, apo-fed-dark; AFL, apo-fed-light; ASD, apo-starved-dark; ASL, apo-starved-light; SFD, sym -fed-dark; SFL, sym -fed-light; SSD, sym -starved-dark; SSL, sym -starved-light.

10.7717/peerj.20851/supp-15 Supplemental Information 15 Two-way ANOVA of the single and interactive effects of feeding, lighting, and symbiotic state on natural-log transformed total host lipids Bolded values show significance.

10.7717/peerj.20851/supp-16 Supplemental Information 16 Tukey HSD pairwise comparisons between treatment groups on the mean of concentration of natural log-transformed total host lipids based on different feeding regimens Bolded values indicate significantly different p-values. Abbreviations: AFD, apo-fed-dark; AFL, apo-fed-light; ASD, apo-starved-dark; ASL, apo-starved-light; SFD, sym -fed-dark; SFL, sym -fed-light; SSD, sym -starved-dark; SSL, sym -starved-light.

10.7717/peerj.20851/supp-17 Supplemental Information 17 Two-way ANOVA of the single and interactive effects of feeding, lighting condition , and symbiotic state on total host proteins. Bolded values indicate significance

10.7717/peerj.20851/supp-18 Supplemental Information 18 Tukey HSD pairwise comparisons between treatment groups on the mean of concentration of total host protein based on different feeding regimens Bolded values indicate significantly different p-values. Abbreviations: AFD, apo-fed-dark; AFL, apo-fed-light; ASD, apo-starved-dark; ASL, apo-starved-light; SFD, sym -fed-dark; SFL, sym -fed-light; SSD, sym -starved-dark; SSL, sym -starved-light.

10.7717/peerj.20851/supp-19 Supplemental Information 19 Two-way ANOVA of the single and interactive effects of feeding condition and symbiotic state on ORO staining. Bolded values indicate significantly different *p*-values

10.7717/peerj.20851/supp-20 Supplemental Information 20 Tukey HSD pairwise comparisons between treatment groups on the mean of concentration of Oil Red O concentration based on different feeding and symbiotic state Abbreviations: SF, symbiotic and fed; SS, symbiotic and starved; AF, aposymbiotic and fed; AS, aposymbiotic and starved. Bolded values indicate significantly different *p*-values.

## Additional Information and Declarations

### Data Availability

The following information was supplied regarding data availability: The data is available at Zenodo: White, E. (2025). Raw data and R files for Heterotrophy and symbiosis affect metabolite provisioning for pedal lacerates in the sea anemone Exaiptasia diaphana. [Data set]. Zenodo. https://doi.org/10.5281/zenodo.15851562.
