# Supplementary material for: Heterotrophy and symbiosis affect energy reserves for pedal lacerates in the sea anemone Exaiptasia diaphana
Source: PeerJ. 2026 Feb 25;14:e20851. doi: 10.7717/peerj.20851 (PMC12949582; doi:10.7717/peerj.20851)
Supplement: Supplemental Information 1 [file peerj-14-20851-s001.docx]

**Supplemental Methods:**

*Comparison of metabolites between natural and artificial G1s*

We hypothesized that naturally-produced G1s would be metabolically provisioned differently than surgically-produced G1s because G0s would supply G1s with extra nutrients for growth and development. For this experiment, symbiotic and aposymbiotic G0s (n=24 each) were plated into 6-well polystyrene plates of FASW. Plates with aposymbiotic G0s were wrapped in aluminum foil. To generate abundant naturally-produced G1s, G0s were maintained at husbandry conditions described in the “*Animal maintenance*” section; however, these G0s were fed brine shrimp four to five times per week for four to six h followed by FASW changes to induce more growth and reproduction. During a one-month period of this feeding regimen, plates were scanned for G1s every 24-72 hours and removed via pipetting into a 1.5 mL microfuge tube. G1s were pelleted by centrifugation at 5000 RPM for five min, and then water was removed before being flash frozen at -80℃ until future experiments were conducted. Naturally-produced G1s of different ages (one to three days post-laceration) were distributed as evenly as possible between replicates to eliminate the effects of age on the study. After at least 200 naturally-produced G1s were collected from both symbiotic and aposymbiotic G0s, surgically-produced G1s were generated and collected from the same G0 anemones. All samples were subjected to the total energy reserves protocol described in Methods. Results from replicates were standardized to host protein concentration and to the number of G1s from each respective replicate to determine the best mode of normalization. We found that host protein concentration and number of G1s per replicate scaled together, suggesting that the later would make a suitable normalization factor (Figure S1-2). When comparing the normalization methods, indexing to number of G1s per replicate did not alter the major observed trends. Therefore, we decided to use number of G1s per replicate as our index so protein could be measured as a separate factor in the total energy reserve assays.

**Supplemental Results:**

*Natural and artificial G1s contain similar metabolite quantities and algal densities*

There were no significant differences in total host carbohydrates or proteins between any of the treatment groups (Figures S3 and S4, Two-way ANOVA, p=0.3746 and p=0.7355, respectively; Tukey HSD) or in symbiont density between natural and artificial G1s (Figure S5, Student’s Two-Sample t-test, p=0.8249). Protein concentrations were similar between treatment groups, suggesting there was no confounding effect of the size of G1s on total energy reserves. Total host lipid quantity significantly differed between test groups based on symbiotic state and G1 type (mode of laceration) (Figure S6, Table S1, Two-way ANOVA, p=.0002 and p=0.0001, respectively). Surgically-produced G1s had higher concentrations of lipids than naturally-produced counterparts and aposymbiotic G1s had higher lipids than symbiotic G1s (Figure S6, Tukey HSD). This suggests that there was a contribution to total lipid concentrations based on type of G1 generation and symbiotic state, though there was not an interactive effect (Two-way ANOVA, p=0.9276).
