# Supplementary figures and images for: Heterotrophy and symbiosis affect energy reserves for pedal lacerates in the sea anemone Exaiptasia diaphana
Source: PeerJ. 2026 Feb 25;14:e20851. doi: 10.7717/peerj.20851 (PMC12949582; doi:10.7717/peerj.20851)

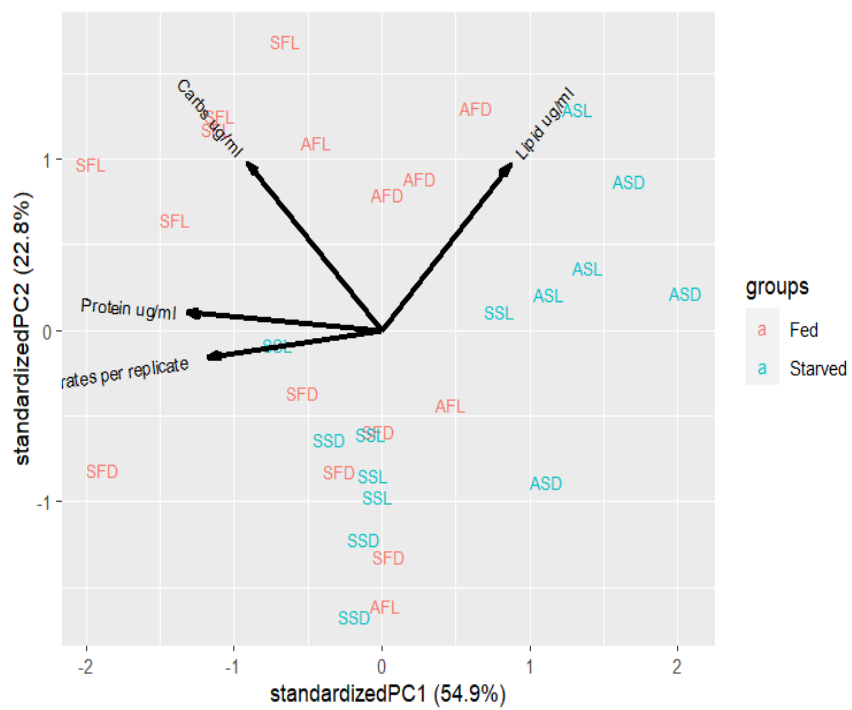

Supplement: Supplemental Information 2 — Abbreviations for test groups are as follows: AFD, aposymbiotic-fed-dark; AFL, aposymbiotic-fed-light; ASD, aposymbiotic-starved-dark; ASL, aposymbiotic-starved-light; SFD, symbiotic-fed-dark; SFL, symbiotic-fed-light; SSD, symbiotic-starved-dark; SSL, symbiotic-starved-light [file peerj-14-20851-s002.pdf]

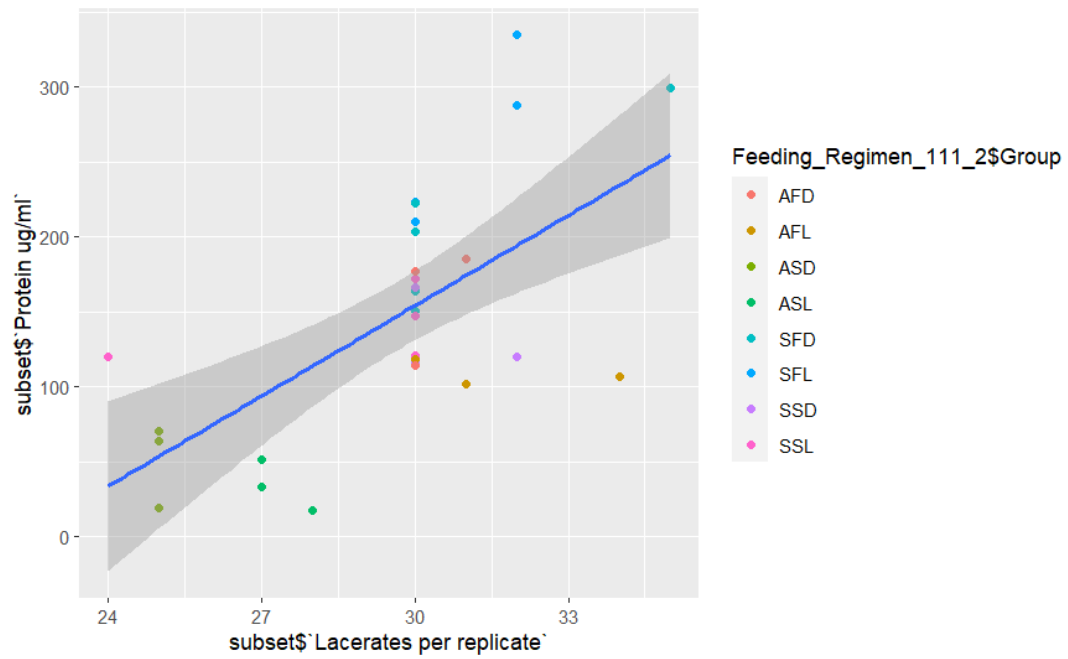

Supplement: Supplemental Information 3 — Y=26.5847 + 0.0206x, R2 = 0.4139, p = 0.0001. Abbreviations for test groups are as follows: AFD, aposymbiotic-fed-dark; AFL, aposymbiotic-fed-light; ASD, aposymbiotic-starved-dark; ASL, aposymbiotic-starved-light; SFD, symbiotic-fed-dark; SFL, symbiotic-fed-light; SSD, symbiotic-starved-dark; SSL, symbiotic-starved-light. [file peerj-14-20851-s003.pdf]

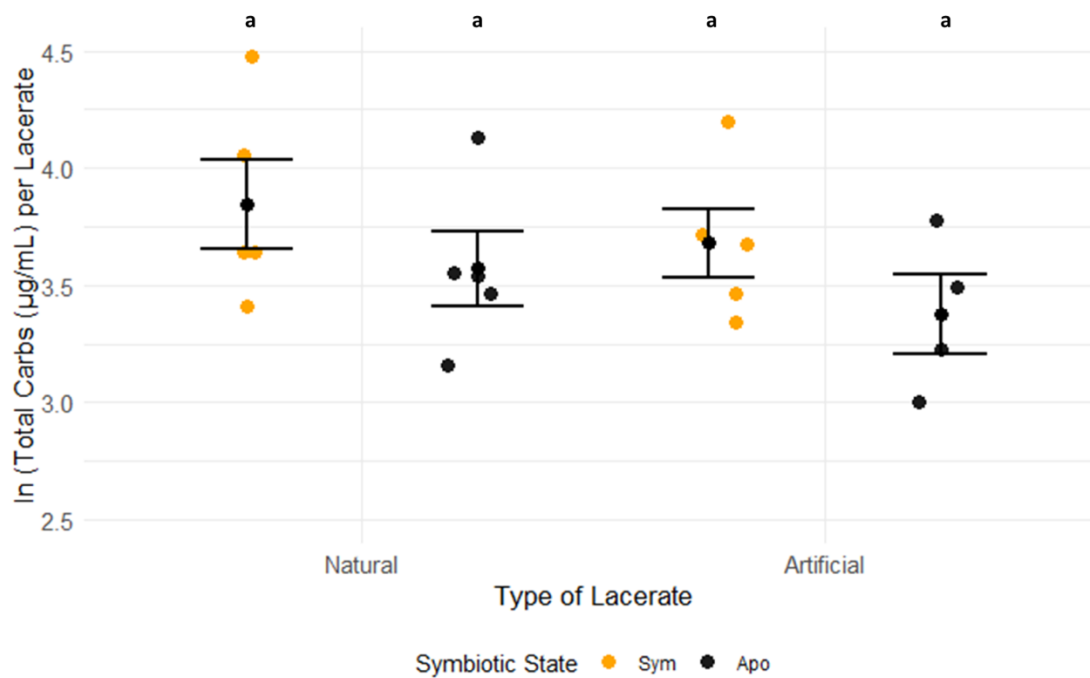

Supplement: Supplemental Information 4 — Black dots and error bars represent group mean +/- standard error, while colored, jittered dots represent individual replicate (n = 30 G1s) optical density values optical run in triplicate. No significant differences were detected between comparisons (Tukey HSD). [file peerj-14-20851-s004.pdf]

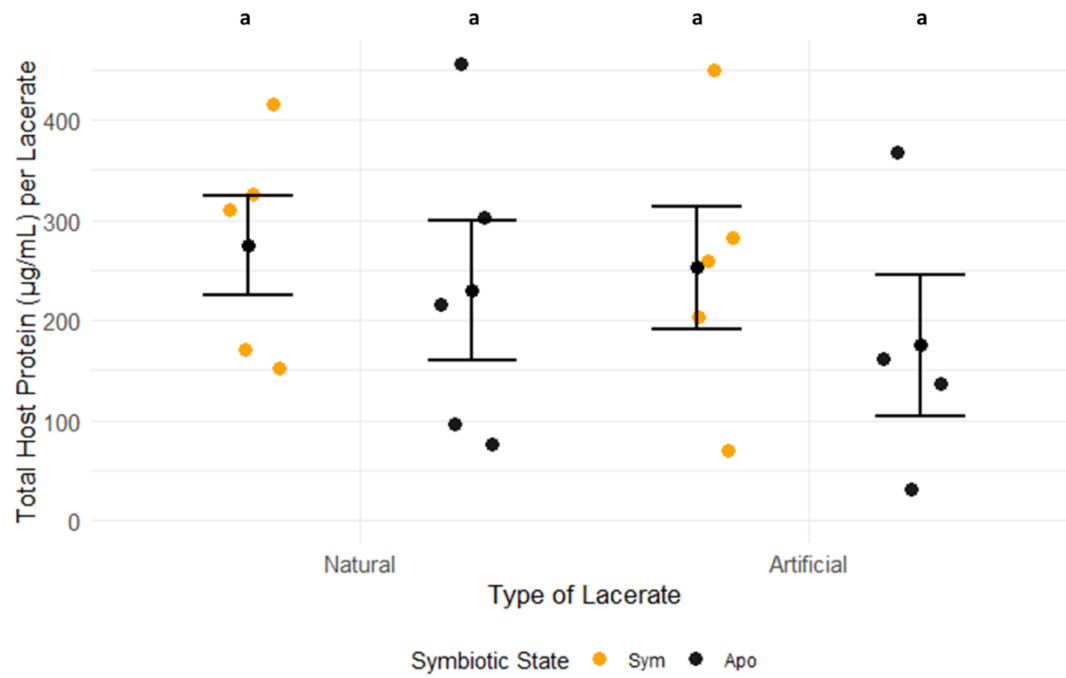

Supplement: Supplemental Information 5 — Black dots and error bars represent the mean concentration +/- standard error for each test group, while colored, jittered dots represent individual replicate (n = 30 G1s) optical density values optical run in triplicate. No significant differences were detected between comparisons (Tukey HSD). [file peerj-14-20851-s005.pdf]

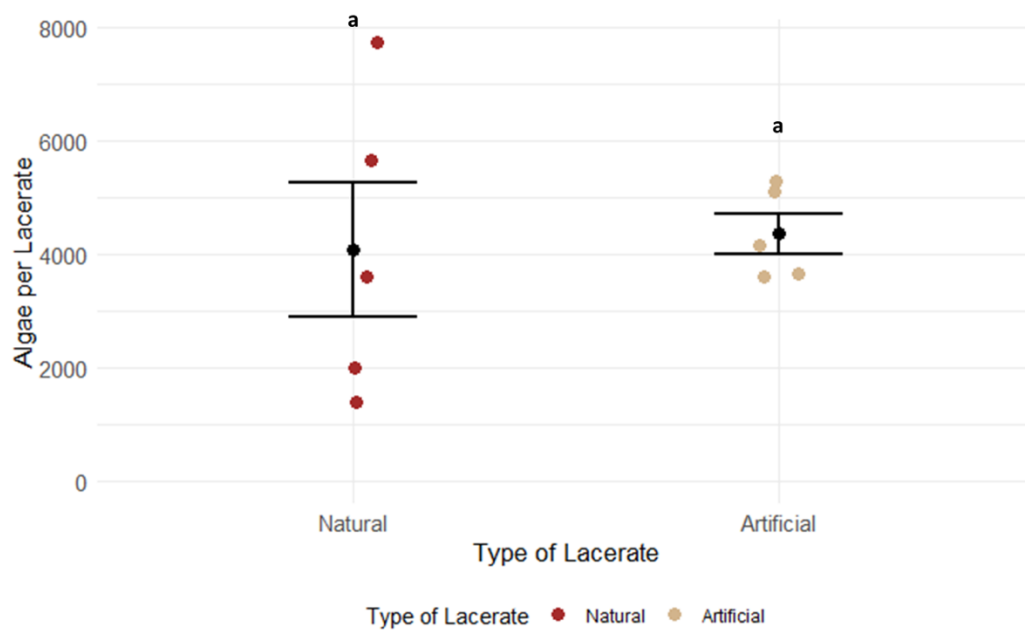

Supplement: Supplemental Information 6 — Black dots and error bars represent the group mean +/- standard error in algal counts between five biological replicates of n = 30 G1s run in triplicate (shown in jittered, colored dots). No significant differences were detected between treatment groups (Student’s two-sample t-test). [file peerj-14-20851-s006.pdf]

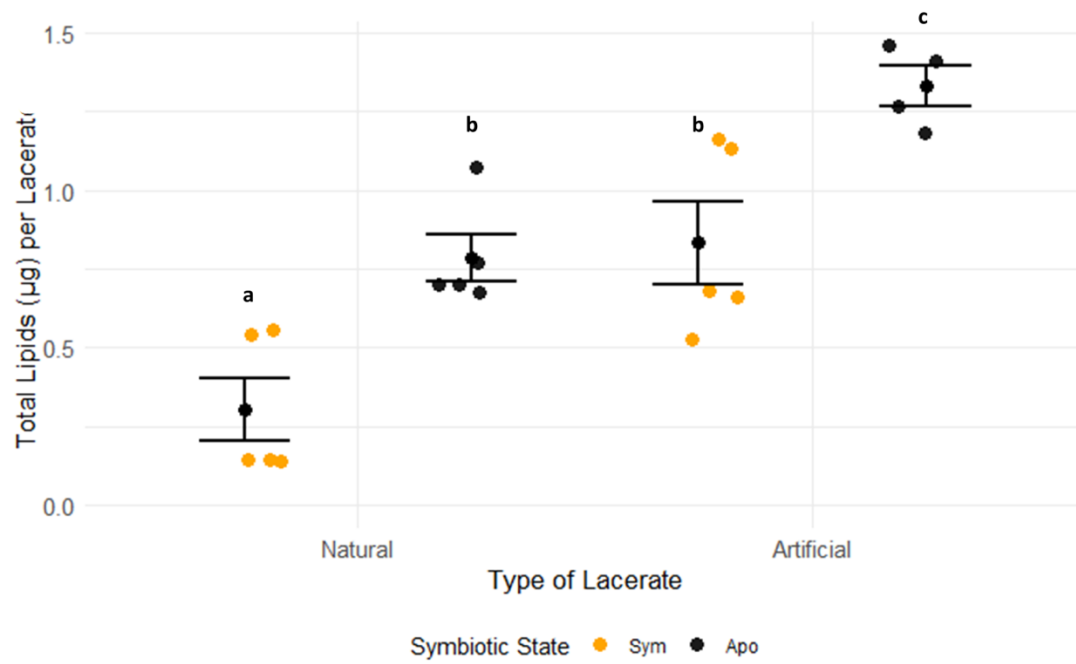

Supplement: Supplemental Information 7 — Black dots and error bars represent group mean +/ standard error, while colored, jittered dots represent individual replicate (n = 30 G1s) optical density values optical run in triplicate. Different letters represent significant pairwise comparisons between test groups differences (Tukey HSD). [file peerj-14-20851-s007.pdf]

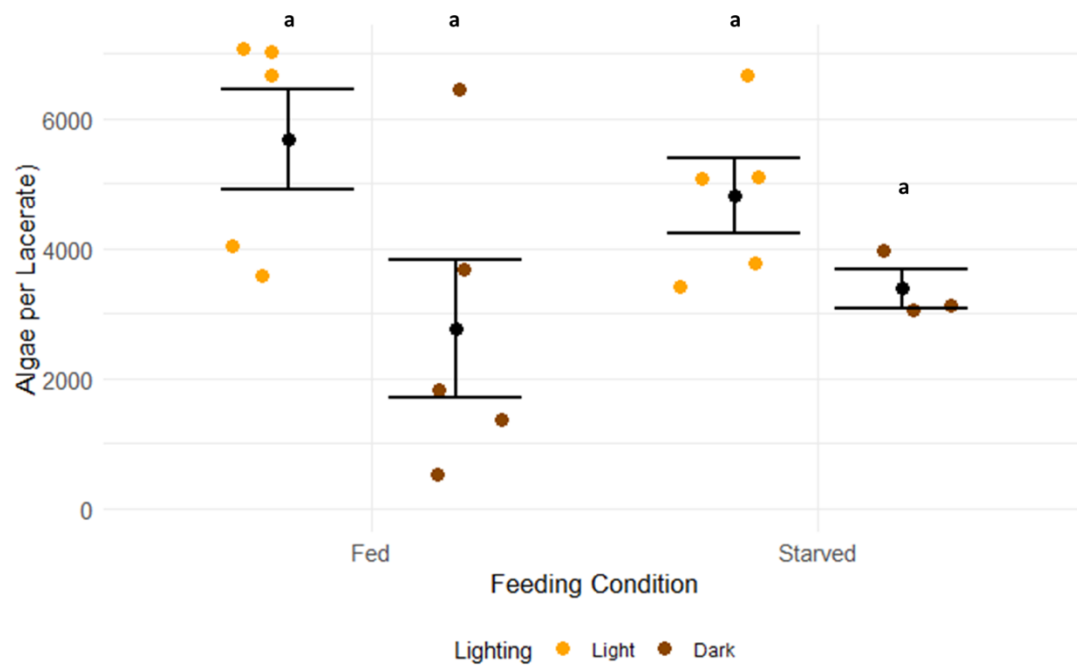

Supplement: Supplemental Information 8 — Black dots and error bars represent the mean concentration +/- standard error for each test group, while colored, jittered dots represent individual biological replicates (n = 30 G1s) of algal counts run in triplicate. No significant differences were detected between comparisons (Tukey HSD post-hoc analysis). [file peerj-14-20851-s008.pdf]
