# Supplementary material for: Heterotrophy and symbiosis affect energy reserves for pedal lacerates in the sea anemone Exaiptasia diaphana
Source: PeerJ. 2026 Feb 25;14:e20851. doi: 10.7717/peerj.20851 (PMC12949582; doi:10.7717/peerj.20851)
Supplement: Supplemental Information 9 — Statistically significant values are bolded. [file peerj-14-20851-s009.docx]

| **Factor** | **df** | **Exact F** | **p-value** |
| --- | --- | --- | --- |
| Type of G1 | 1 | 26.1391 | **0.0001274** |
| Symbiotic state | 1 | 23.9071 | **0.0001963** |
| Type of G1:Symbiotic state | 1 | 0.0086 | 0.9275490 |
