# Supplementary material for: Heterotrophy and symbiosis affect energy reserves for pedal lacerates in the sea anemone Exaiptasia diaphana
Source: PeerJ. 2026 Feb 25;14:e20851. doi: 10.7717/peerj.20851 (PMC12949582; doi:10.7717/peerj.20851)
Supplement: Supplemental Information 10 — Abbreviations: AA, artificial and aposymbiotic; AS, Artificial and symbiotic; NA, natural and aposymbiotic; NS, natural and symbiotic. Bolded values indicate significantly different p-values (p < 0.05). [file peerj-14-20851-s010.docx]

| **Group** | **Difference** | **Lower Bound** | **Upper Bound** | **p-value** |
| --- | --- | --- | --- | --- |
| AS-AA | -0.498 | -0.919 | -0.078 | **0.017** |
| NA-AA | -0.547 | -0.967 | -0.127 | **0.0091** |
| NS-AA | -1.027 | -1.448 | -0.607 | **0.00002** |
| NA-AS | -0.048 | -0.4448 | 0.347 | 0.984 |
| NS-AS | -0.528 | -0.925 | -0.1328 | **0.0072** |
| NS-NA | -0.48 | -0.876 | -0.876 | **0.015** |
