# Supplementary material for: Heterotrophy and symbiosis affect energy reserves for pedal lacerates in the sea anemone Exaiptasia diaphana
Source: PeerJ. 2026 Feb 25;14:e20851. doi: 10.7717/peerj.20851 (PMC12949582; doi:10.7717/peerj.20851)
Supplement: Supplemental Information 11 — Bolded values indicate statistical significance. [file peerj-14-20851-s011.docx]

| **Factor** | **df** | **Exact f** | **p-value** |
| --- | --- | --- | --- |
| Feeding condition | 1 | 17.1066 | **0.000127** |
| Lighting condition | 1 | 22.8104 | **1.45e-05** |
| Symbiotic state | 1 | 5.3525 | **0.0246** |
| Feeding condition:Light condition | 1 | 4.9559 | **0.03027** |
| Feeding condition:Symbiotic state | 1 | 13.6817 | **0.000516** |
| Light condition:Symbiotic state | 1 | 6.2484 | **0.0156** |
| Feeding condition:Light condition:Symbiotic state | 1 | 2.9694 | 0.091 |
