# Supplementary material for: Heterotrophy and symbiosis affect energy reserves for pedal lacerates in the sea anemone Exaiptasia diaphana
Source: PeerJ. 2026 Feb 25;14:e20851. doi: 10.7717/peerj.20851 (PMC12949582; doi:10.7717/peerj.20851)
Supplement: Supplemental Information 12 — Bolded values indicate significantly different p-values. Abbreviations: AFD, apo-fed-dark; AFL, apo-fed-light; ASD, apo-starved-dark; ASL, apo-starved-light; SFD, sym-fed-dark; SFL, sym-fed-light; SSD, sym-starved-dark; SSL, sym-starved-light. [file peerj-14-20851-s012.docx]

| **Group** | **Difference** | **Lower Bound** | **Upper Bound** | **p-value** |
| --- | --- | --- | --- | --- |
| AFL-AFD | 75.35237 | -197.61157 | 348.316306 | 0.9874369 |
| ASD-AFD | -331.77039 | -590.35757 | -73.183204 | **0.0039355** |
| ASL-AFD | -285.86061 | -569.12868 | -2.592549 | **0.0464469** |
| SFD-AFD | -398.72889 | -652.09155 | -145.366229 | **0.0001921** |
| SFL-AFD | 27.70486 | -255.5632 | 310.972927 | 0.9999851 |
| SSD-AFD | -306.07603 | -571.04904 | -41.103018 | **0.0132025** |
| SSL-AFD | -188.86291 | -447.45009 | 69.72427 | 0.3106203 |
| ASD-AFL | -407.12275 | -654.37939 | -159.866117 | **0.0000865** |
| ASL-AFL | -361.21298 | -634.17692 | -88.249045 | **0.0026417** |
| SFD-AFL | -474.08126 | -715.86867 | -232.293847 | **0.0000025** |
| SFL-AFL | -47.64751 | -320.61144 | 225.316431 | 0.9992752 |
| SSD-AFL | -381.4284 | -635.35597 | -127.500827 | **0.0004155** |
| SSL-AFL | -264.21528 | -511.47192 | -16.958642 | **0.0283345** |
| ASL-ASD | 45.90977 | -212.67741 | 304.496953 | 0.9991907 |
| SFD-ASD | -66.9585 | -292.38958 | 158.472575 | 0.9808455 |
| SFL-ASD | 359.47525 | 100.88807 | 618.062429 | **0.0013421** |
| SSD-ASD | 25.69436 | -212.71125 | 264.099957 | 0.9999711 |
| SSL-ASD | 142.90747 | -88.37993 | 374.19488 | 0.5245274 |
| SFD-ASL | -112.86828 | -366.23093 | 140.494384 | 0.8506951 |
| SFL-ASL | 313.56548 | 30.29741 | 596.833541 | **0.0203191** |
| SSD-ASL | -20.21542 | -285.18843 | 244.757595 | 0.9999973 |
| SSL-ASL | 96.9977 | -161.58948 | 355.584884 | 0.933153 |
| SFL-SFD | 426.43375 | 173.07109 | 679.79641 | **0.0000577** |
| SSD-SFD | 92.65286 | -140.0756 | 325.381322 | 0.910569 |
| SSDL-SFD | 209.86598 | -15.56510 | 435.297056 | 0.0852785 |
| SSD-SFL | -333.78089 | -598.7539 | -68.807881 | **0.0049398** |
| SSL-SFL | -216.56777 | -475.15495 | 42.019407 | 0.1640177 |
| SSL-SSD | 117.21312 | -121.19248 | 355.61872 | 0.7761389 |
