# Supplementary material for: Heterotrophy and symbiosis affect energy reserves for pedal lacerates in the sea anemone Exaiptasia diaphana
Source: PeerJ. 2026 Feb 25;14:e20851. doi: 10.7717/peerj.20851 (PMC12949582; doi:10.7717/peerj.20851)
Supplement: Supplemental Information 13 — Bolded values indicate statistical significance. [file peerj-14-20851-s013.docx]

| **Factor** | **df** | **Exact f** | **p-value** |
| --- | --- | --- | --- |
| Feeding condition | 1 | 6.220 | **0.02063** |
| Lighting condition | 1 | 23.339 | **7.94e-05** |
| Symbiotic state | 1 | 6.991 | **0.01482** |
| Feeding condition:Light condition | 1 | 3.408 | 0.07838 |
| Feeding condition:Symbiotic state | 1 | 2.831 | 0.10658 |
| Light condition:Symbiotic state | 1 | 9.845 | **0.00478** |
| Feeding condition:Light condition:Symbiotic state | 1 | 8.467 | **0.00812** |
