# Supplementary material for: Heterotrophy and symbiosis affect energy reserves for pedal lacerates in the sea anemone Exaiptasia diaphana
Source: PeerJ. 2026 Feb 25;14:e20851. doi: 10.7717/peerj.20851 (PMC12949582; doi:10.7717/peerj.20851)
Supplement: Supplemental Information 14 — Bolded values indicate significantly different p-values. Abbreviations: AFD, apo-fed-dark; AFL, apo-fed-light; ASD, apo-starved-dark; ASL, apo-starved-light; SFD, sym -fed-dark; SFL, sym -fed-light; SSD, sym -starved-dark; SSL, sym -starved-light. [file peerj-14-20851-s014.docx]

| **Group** | **Difference** | **Lower Bound** | **Upper Bound** | **p-value** |
| --- | --- | --- | --- | --- |
| AFL-AFD | -1.559 | -11.663 | 8.5436 | 0.999 |
| ASD-AFD | -3.376 | -13.479 | 6.7269 | 0.946 |
| ASL-AFD | 0.483 | -9.619 | 10.5869 | 0.999 |
| SFD-AFD | -2.146 | -11.1835 | 6.89 | 0.9917 |
| SFL-AFD | 12.673 | 3.636 | 21.7099 | **0.0024** |
| SSD-AFD | -2.092 | -12.195 | 8.011 | 0.996 |
| SSL-AFD | 1.9109 | -7.125 | 10.947 | 0.9959 |
| ASD-AFL | -1.816 | -11.92 | 8.286 | 0.998 |
| ASL-AFL | 2.043 | -8.06 | 12.1467 | 0.996 |
| SFD-AFL | -0.586 | -9.6236 | 8.4499 | 0.999 |
| SFL-AFL | 14.2329 | 5.196 | 23.2697 | **0.0006** |
| SSD-AFL | -0.532 | -10.6357 | 9.5711 | 0.999 |
| SSL-AFL | 3.4707 | -5.566 | 12.5075 | 0.8959 |
| ASL-ASD | 3.86 | -6.243 | 13.9634 | 0.8983 |
| SFD-ASD | 1.229 | -7.8069 | 10.266 | 0.999 |
| SFL-ASD | 16.049 | 7.0128 | 25.086 | **0.0001** |
| SSD-ASD | 1.284 | -8.819 | 11.387 | 0.999 |
| SSL-ASD | 5.287 | -3.749 | 14.324 | 0.5319 |
| SFD-ASL | -2.63 | -11.666 | 6.4066 | 0.9739 |
| SFL-ASL | 12.189 | 3.1528 | 21.226 | **0.0036** |
| SSD-ASL | -2.575 | -12.679 | 7.527 | 0.987 |
| SSL-ASL | 1.427 | -7.609 | 10.464 | 0.999 |
| SFL-SFD | 14.819 | 6.9937 | 22.645 | **0.00005** |
| SSD-SFD | 0.054 | -8.982 | 9.091 | 1.000 |
| SSDL-SFD | 4.057 | -3.768 | 11.8837 | 0.668 |
| SSD-SFL | -14.765 | -23.802 | -5.728 | **0.00039** |
| SSL-SFL | -10.762 | -18.588 | -2.936 | **0.0029** |
| SSL-SSD | 4.003 | -5.0337 | 13.039 | 0.8101 |
