# Supplementary material for: Heterotrophy and symbiosis affect energy reserves for pedal lacerates in the sea anemone Exaiptasia diaphana
Source: PeerJ. 2026 Feb 25;14:e20851. doi: 10.7717/peerj.20851 (PMC12949582; doi:10.7717/peerj.20851)
Supplement: Supplemental Information 15 — Bolded values show significance. [file peerj-14-20851-s015.docx]

| **Factor** | **df** | **Exact F** | **p-value** |
| --- | --- | --- | --- |
| Feeding condition | 1 | 0.6574 | 0.7513 |
| Lighting condition | 1 | 1.0005 | 0.7565 |
| Symbiotic state | 1 | 48.7794 | **2.837e-06** |
| Feeding condition:Light condition | 1 | 1.7690 | 0.17209 |
| Feeding condition:Symbiotic state | 1 | 4.2752 | 0.06755 |
| Light condition:Symbiotic state | 1 | 5.7463 | **0.03242** |
| Feeding condition:Light condition:Symbiotic state | 1 | 2.3248 | 0.2392 |
