# Supplementary material for: Heterotrophy and symbiosis affect energy reserves for pedal lacerates in the sea anemone Exaiptasia diaphana
Source: PeerJ. 2026 Feb 25;14:e20851. doi: 10.7717/peerj.20851 (PMC12949582; doi:10.7717/peerj.20851)
Supplement: Supplemental Information 16 — Bolded values indicate significantly different p-values. Abbreviations: AFD, apo-fed-dark; AFL, apo-fed-light; ASD, apo-starved-dark; ASL, apo-starved-light; SFD, sym -fed-dark; SFL, sym -fed-light; SSD, sym -starved-dark; SSL, sym -starved-light. [file peerj-14-20851-s016.docx]

| **Group** | **Difference** | **Lower Bound** | **Upper Bound** | **p-value** |
| --- | --- | --- | --- | --- |
| AFL-AFD | -0.634 | -1.4709 | 0.202 | 0.23347 |
| ASD-AFD | -0.11 | -0.946 | 0.726 | 0.999 |
| ASL-AFD | -0.0309 | -0.867 | 0.805 | 1.000 |
| SFD-AFD | -0.9105 | -1.658 | -0.162 | **0.0101** |
| SFL-AFD | -0.755 | -1.5035 | -0.007 | **0.0467** |
| SSD-AFD | -1.196 | -2.0335 | -0.36046 | **0.0019** |
| SSL-AFD | -0.886 | -1.635 | -0.138 | 0.128 |
| ASD-AFL | 0.524 | -0.3125 | 1.3605 | 0.449 |
| ASL-AFL | 0.6035 | -0.233 | 1.44 | 0.285 |
| SFD-AFL | -0.276 | -1.024 | 0.47207 | 0.913 |
| SFL-AFL | -0.1209 | -0.869 | 0.627 | 0.999 |
| SSD-AFL | -0.5625 | -1.399 | 0.2739 | 0.364 |
| SSL-AFL | -0.25245 | -1.0006 | 0.495 | 0.9437 |
| ASL-ASD | 0.07948 | -0.757 | 0.916 | 0.999 |
| SFD-ASD | -0.80015 | -1.548 | -0.0519 | **0.0304** |
| SFL-ASD | -0.6449 | -1.393 | 0.103 | 0.125 |
| SSD-ASD | -1.086 | -1.923 | -0.25007 | **0.0054** |
| SSL-ASD | -0.776 | -1.5246 | -0.02825 | **0.038** |
| SFD-ASL | -0.879 | -1.627 | -0.131 | **0.0138** |
| SFL-ASL | -0.72447 | -1.4726 | 0.0237 | 0.062 |
| SSD-ASL | -1.166 | -2.0026 | -0.329 | **0.0025** |
| SSL-ASL | -0.855 | -1.604 | -0.1077 | **0.0175** |
| SFL-SFD | 0.155 | -0.4928 | 0.803 | 0.991 |
| SSD-SFD | -0.286 | -1.0346 | 0.4617 | 0.897 |
| SSDL-SFD | 0.0236 | -0.624 | 0.6716 | 1.000 |
| SSD-SFL | -0.4416 | -1.189 | 0.306 | 0.5215 |
| SSL-SFL | -0.131 | -0.779 | 0.51648 | 0.996 |
| SSL-SSD | 0.31 | -0.438 | 1.058 | 0.8549 |
