# Supplementary material for: Heterotrophy and symbiosis affect energy reserves for pedal lacerates in the sea anemone Exaiptasia diaphana
Source: PeerJ. 2026 Feb 25;14:e20851. doi: 10.7717/peerj.20851 (PMC12949582; doi:10.7717/peerj.20851)
Supplement: Supplemental Information 17 [file peerj-14-20851-s017.docx]

| **Factor** | **df** | **Exact F** | **p-value** |
| --- | --- | --- | --- |
| Feeding condition | 1 | 34.811 | **6.15e-06** |
| Lighting condition | 1 | 0.058 | 0.812 |
| Symbiotic state | 1 | 36.022 | **4.85e-06** |
| Feeding condition:Light condition | 1 | 0.047 | 0.83 |
| Feeding condition:Symbiotic state | 1 | 0.013 | 0.9113 |
| Light condition:Symbiotic state | 1 | 4.458 | **0.0463** |
| Feeding condition:Light condition:Symbiotic state | 1 | 0.833 | 0.3712 |
