# Supplementary material for: Heterotrophy and symbiosis affect energy reserves for pedal lacerates in the sea anemone Exaiptasia diaphana
Source: PeerJ. 2026 Feb 25;14:e20851. doi: 10.7717/peerj.20851 (PMC12949582; doi:10.7717/peerj.20851)
Supplement: Supplemental Information 18 — Bolded values indicate significantly different p-values. Abbreviations: AFD, apo-fed-dark; AFL, apo-fed-light; ASD, apo-starved-dark; ASL, apo-starved-light; SFD, sym -fed-dark; SFL, sym -fed-light; SSD, sym -starved-dark; SSL, sym -starved-light. [file peerj-14-20851-s018.docx]

| **Group** | **Difference** | **Lower Bound** | **Upper Bound** | **p-value** |
| --- | --- | --- | --- | --- |
| AFL-AFD | -1.7665 | -5.298 | 1.765 | 0.70515 |
| ASD-AFD | -3.182 | -6.714 | 0.349 | 0.0979 |
| ASL-AFD | -3.973 | -7.505 | -0.441 | **0.0202** |
| SFD-AFD | 1.415 | -1.7438 | 4.575 | 0.8016 |
| SFL-AFD | 2.5425 | -0.616 | 5.7017 | 0.1787 |
| SSD-AFD | -0.8289 | -4.3609 | 2.703 | 0.9923 |
| SSL-AFD | -0.506 | -3.666 | 2.652 | 0.999 |
| ASD-AFL | -1.415 | -4.947 | 2.116 | 0.874 |
| ASL-AFL | -2.206 | -5.738 | 1.325 | 0.4527 |
| SFD-AFL | 3.1818 | 0.0226 | 6.341 | **0.0475** |
| SFL-AFL | 4.309 | 1.149 | 7.468 | **0.003** |
| SSD-AFL | 0.937 | -2.594 | 4.469 | 0.984 |
| SSL-AFL | 1.259 | -1.899 | 4.418 | 0.877 |
| ASL-ASD | -0.791 | -4.323 | 2.7409 | 0.994 |
| SFD-ASD | 4.597 | 1.438 | 7.756 | **0.0016** |
| SFL-ASD | 5.7249 | 2.565 | 8.884 | **0.0001** |
| SSD-ASD | 2.353 | -1.178 | 5.885 | 0.3755 |
| SSL-ASD | 2.675 | -0.4837 | 5.8346 | 0.1384 |
| SFD-ASL | 5.388 | 2.229 | 8.547 | **0.0002** |
| SFL-ASL | 6.515 | 3.356 | 9.675 | **0.00001** |
| SSD-ASL | 3.14446 | -0.387 | 6.676 | 0.105 |
| SSL-ASL | 3.466 | 0.307 | 6.625 | **0.0248** |
| SFL-SFD | 1.127 | -1.608 | 3.863 | 0.858 |
| SSD-SFD | -2.44 | -5.403 | 0.9149 | 0.302 |
| SSDL-SFD | -1.922 | -4.658 | 0.8137 | 0.314 |
| SSD-SFL | -3.3715 | -6.5306 | -0.212 | **0.0309** |
| SSL-SFL | -3.049 | -5.785 | -0.3135 | **0.0218** |
| SSL-SSD | 0.322 | -2.837 | 3.481 | 0.999 |
