# Supplementary material for: Heterotrophy and symbiosis affect energy reserves for pedal lacerates in the sea anemone Exaiptasia diaphana
Source: PeerJ. 2026 Feb 25;14:e20851. doi: 10.7717/peerj.20851 (PMC12949582; doi:10.7717/peerj.20851)
Supplement: Supplemental Information 19 [file peerj-14-20851-s019.docx]

| **Factor** | **df** | **Exact F** | **p-value** |
| --- | --- | --- | --- |
| Feeding Condition | 1 | 0.043 | 0.8385 |
| Symbiotic state | 1 | 61.501 | **1.1e-06** |
| Feeding Condition:Symbiotic state | 1 | 3.546 | 0.0792 |
