# Supplementary material for: Heterotrophy and symbiosis affect energy reserves for pedal lacerates in the sea anemone Exaiptasia diaphana
Source: PeerJ. 2026 Feb 25;14:e20851. doi: 10.7717/peerj.20851 (PMC12949582; doi:10.7717/peerj.20851)
Supplement: Supplemental Information 20 — Abbreviations: SF, symbiotic and fed; SS, symbiotic and starved; AF, aposymbiotic and fed; AS, aposymbiotic and starved. Bolded values indicate significantly different p-values. [file peerj-14-20851-s020.docx]

| **Group** | **Difference** | **Lower Bound** | **Upper Bound** | **p-value** |
| --- | --- | --- | --- | --- |
| AS-AF | 0.5338 | -0.7344 | 1.802 | 0.628 |
| SF-AF | 2.903 | 1.707 | 4.099 | **0.00002** |
| SS-AF | 2.298 | 1.1026 | 3.494 | **0.0003** |
| SF-AS | 2.369 | 1.101 | 3.637 | **0.0004** |
| SS-AS | 1.7645 | 0.496 | 3.0328 | **0.0055** |
| SS-SF | -0.6049 | -1.8007 | 0.5908 | 0.485 |
